# Supplementary material for: Elevational differences in hydrogeomorphic disturbance regime influence sediment residence times within mountain river corridors
Source: Nat Commun. 2019 May 20;10:2221. doi: 10.1038/s41467-019-09864-w (PMC6527556; doi:10.1038/s41467-019-09864-w)
Supplement: Supplementary file 1 — Supplementary Information [file 41467_2019_9864_MOESM1_ESM.pdf]

Elevational differences in hydrogeomorphic disturbance regime influence sediment residence times within mountain river corridors

Nicholas A. Sutfin and Ellen Wohl

Supplementary Information

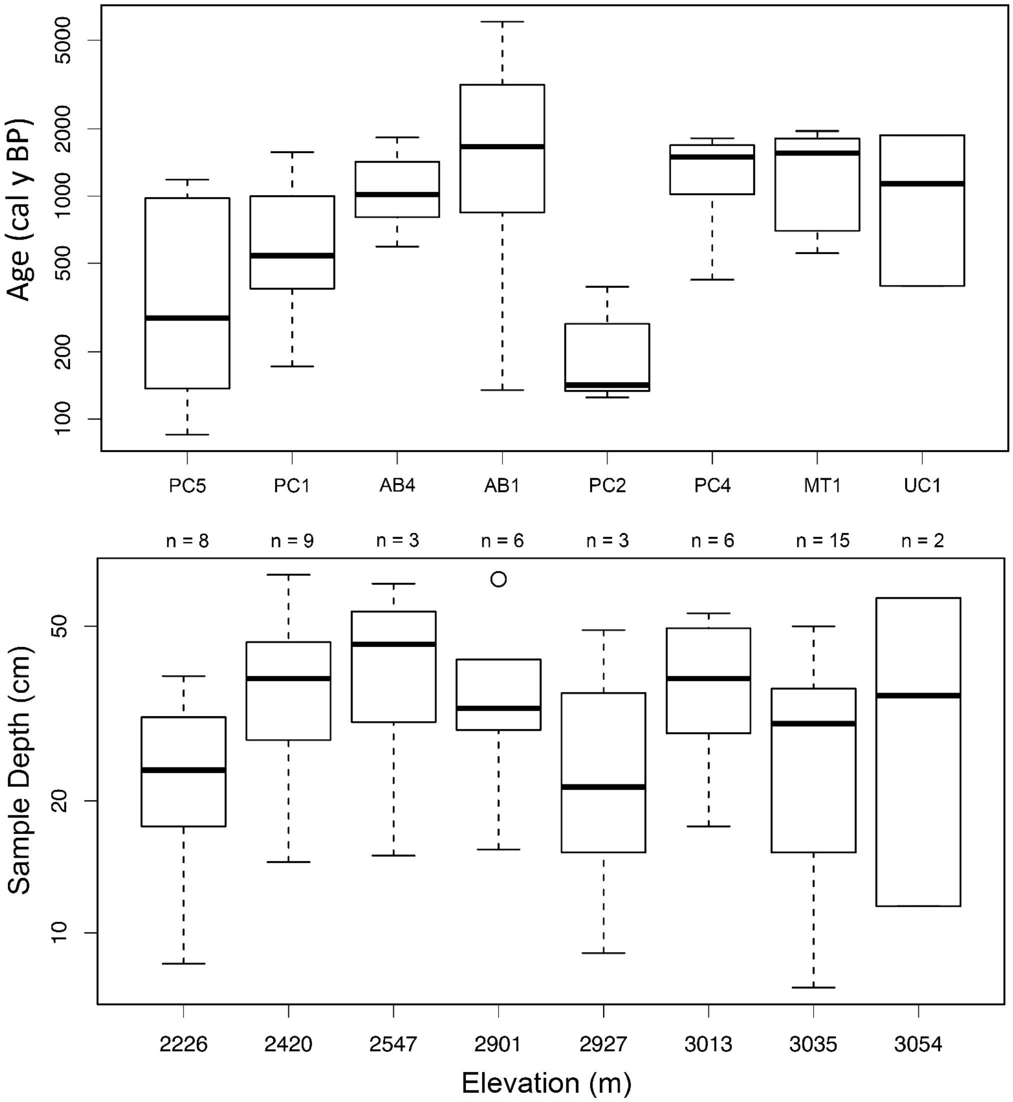

Supplementary Figure S1. Boxplots of charcoal sample weighted mean radiocarbon ages and depth. Plots for each study reach illustrate variability in sample depth and illustrate show that younger mean ages do not solely represent charcoal samples from more shallow depths. Thick lines represent the median (50th percentile), upper and lower edges of the boxes represent the 25th and 75th percentile. Whiskers extend to the minimum and maximum values within 1.5 times the interquartile range and open circles are outliers exceeding that range. Source data are provided as a Source Data file.

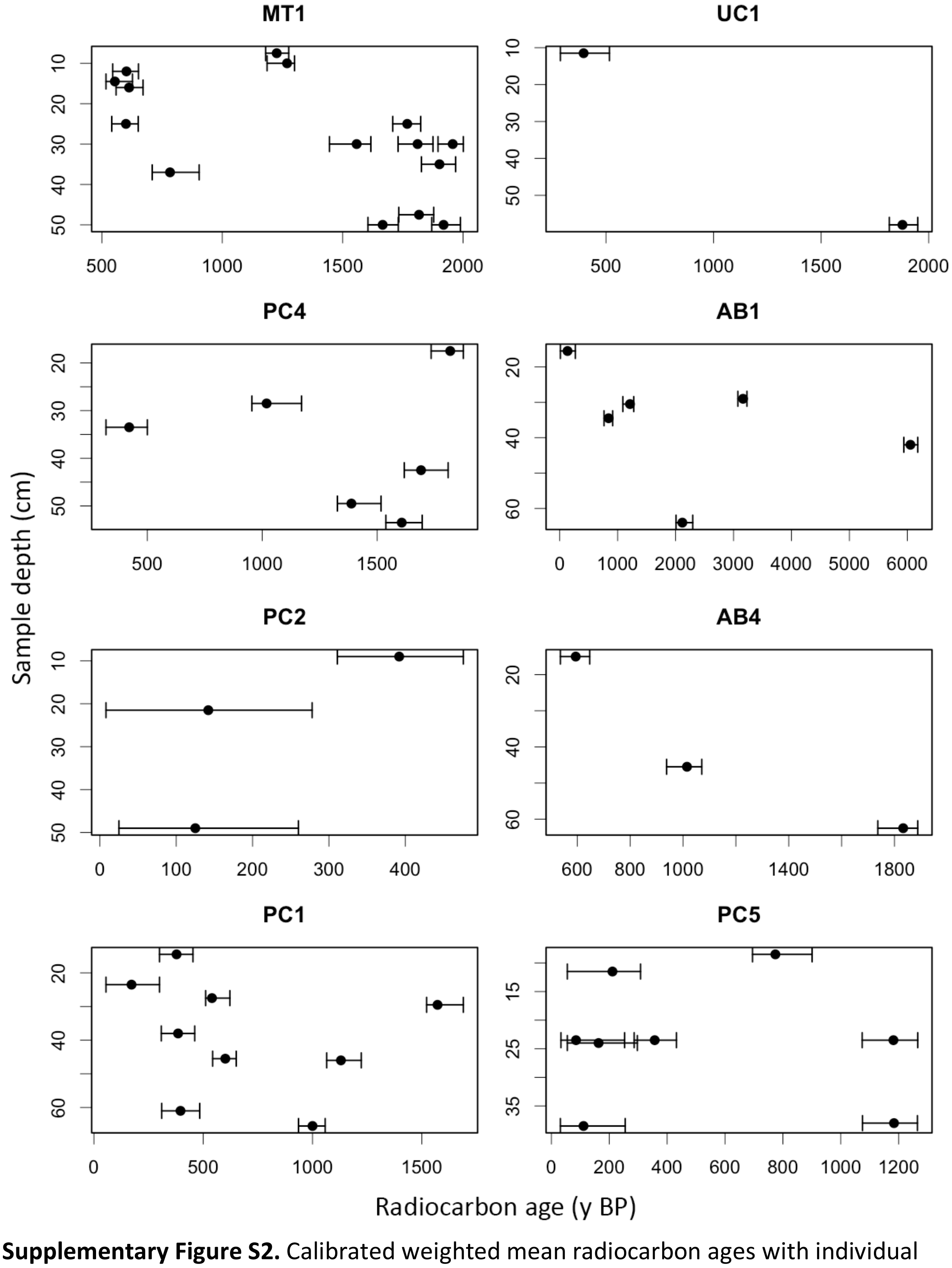

Supplementary Figure S2. Calibrated weighted mean radiocarbon ages with individual sample depth along each study reach. Mean ages represent years before 1950 and error bars indicate the 95% confidence level for age estimates. Source data are provided as a Source Data file.

Supplementary Table S1. Uncalibrated and calibrated ^{14}C ages.

Sample		Un-calibrated sample analysis			Calibrated ages (y BP)				
Sample ID	Ave depth (cm)	^{14}C age (y BP)	^{14}C Age SE (y)	delta ^{13}C (per mil)	Max	Min	Weighted Mean	Sigma	Median
AB1S2.2	34.5	909	23	-20.2	914	765	844	44	852
AB1S3.1	15.5	121	23	-23.1	270	12	135	78	114
AB1S4.2	29	2989	23	-21.6	3232	3075	3164	46	3170
AB1S4.3	42	5267	28	-18.8	6179	5939	6053	70	6052
AB1S6.2	30.5	1256	23	-21.2	1278	1090	1214	38	1220
AB1S8.3	64	2131	23	-20.7	2296	2008	2117	60	2115
AB4S10.4	62.5	1887	24	-15.4	1888	1737	1833	37	1840
AB4S12	15	581	25	-15.6	647	536	594	33	605
AB4S6.3	45.5	1110	29	-19.3	1071	938	1015	37	1013
MT1S12	30	1654	24	-18.7	1617	1445	1558	33	1555
MT1S14	30	2009	23	-24.1	2001	1896	1957	29	1958
MT1S16	30	1872	25	-23.2	1875	1730	1811	43	1820
MT1S17	47.5	1876	25	-18.4	1878	1733	1817	42	1826
MT1S2.0	10	1332	22	-21.1	1300	1186	1269	28	1278
MT1S2.1	7.5	1274	24	-18.9	1276	1180	1226	32	1229
MT1S2.2	25	1831	23	-19.6	1824	1708	1768	35	1769
MT1S2.3	35	1952	23	-17.1	1969	1827	1902	28	1901
MT1S20	50	1761	22	-22.1	1731	1605	1666	35	1662
MT1S3.0	14.5	538	22	-17.4	627	517	554	33	541
MT1S4.1	16	657	27	-17.9	671	559	613	38	601
MT1S5.0	12	602	22	-18.5	652	545	602	30	604
MT1S6.0	25	595	23	-19.4	651	541	600	31	605
MT1S6.1	37	870	25	-21.4	904	709	783	47	772
MT1S7.2	50	1968	26	-17.6	1989	1870	1919	31	1917
PC1LS10.3	45.5	599	23	-24.5	651	543	601	31	604
PC1LS10.4	61	342	32	-14.5	484	310	396	52	392
PC1LS8.3	46	1208	21	-22.3	1223	1065	1130	40	1128
PC1RS1.1	14.5	300	23	-24.9	453	300	378	44	390
PC1RS1.3	38	324	22	-22.8	461	308	385	44	386
PC1RS5.2	29.5	1665	28	-24.9	1690	1522	1572	38	1567
PC1RS5.5	65.5	1089	29	-17.7	1058	936	1000	36	995
PC1S6.2	27.5	524	23	-23.9	622	511	540	25	534
PC1S7.2	23.5	201	26	-28.7	300	…	172	93	176
PC2S1.3	49	84	28	-20.7	260	25	125	77	105
PC2S3.1	21.5	134	30	-18.4	278	8	142	81	134
PC2S4.1	9	337	27	-22.6	476	311	392	49	388
PC4S3.3	42.5	1780	22	-22.2	1809	1618	1691	47	1697
PC4S3.4	53.5	1695	31	-22.9	1696	1538	1607	44	1598
PC4S4.1	17.5	1875	21	-24.8	1875	1735	1818	40	1827
PC4S6.2	33.5	370	21	-20.3	500	320	421	58	445
PC4S6.3	49.5	1503	25	-20.8	1517	1327	1388	39	1382
PC4S7.2	28.5	1116	28	-22.8	1171	955	1019	38	1017
PC5S1.3	38	1240	27	-19.9	1265	1075	1184	54	1197
PC5S10.1	8.5	861	29	-27.6	901	695	774	47	765
PC5S10.2	23.5	279	24	-23.4	432	286	357	56	370
PC5S6.2	23.5	48	20	-21.1	253	33	85	66	53
PC5S6.3	38.5	68	21	-24.5	255	31	111	76	69
PC5S8.1	11.5	225	22	-21.7	308	…	211	88	185
PC5S8.2	24	181	28	-14.6	297	…	163	89	180
PC5S9.2	23.5	1240	29	…	1266	1074	1182	55	1192
UC1S6.4	58	1931	31	-22.5	1949	1817	1878	37	1879
UC1S8.1	11.5	350	69	-25	516	288	396	75	399

Sample depths, uncalibrated radiocarbon ages (where SE is standard error), and calibrated ages from OxCal where the minimum (Min) and maximum (Max) represent the 95.4% confidence level. Additional Oxcal output includes the calibrated weighted mean, the associated error of that mean estimate (sigma), and median age.

Supplementary Table S2. Basic statistics for measured sediment depths.

ReachID	n	Min	Max	Mean	SD	SE
CCKUC1	85	0	100	27.6	25.3	2.7
HCKPC4	100	0	71	28.0	21.9	2.2
NSVAB1	181	0	170	36.7	33.7	2.5
NSVAB4	440	0	154	55.5	35.2	1.7
NSVMT1	144	0	175	17.2	25.5	2.1
NSVPC1	123	0	85	20.2	22.7	2.0
NSVPC5	91	0	97	22.7	24.5	2.6
OCKPC2	82	0	72	14.3	15.8	1.7

Values represent measured depths (cm) of fine floodplain sediment along each of the eight field study site and n is the sample size.

Supplementary Table S3. Correlations for predictor variables, mean ^{14}C age, and sediment flux.

	Elevation (m)	Valley Confinement (m m^{-1})	Stream Gradient, Slope (m m^{-1})	Stream Power (W m^{-1})	Unit Stream Power (W m^{-2})	Mean Depth (cm)	Mean Age (cal y BP)	Sediment Flux (kg yr^{-1} m^{-1})
Elevation (m)	1						**Y**	**N**
Valley Confinement (m m^{-1})	-0.63	1					**Y**	**Y**
Stream Gradient, Slope (m m^{-1})	0.61	-0.06	1				**N**	**Y**
Stream Power (W m^{-1})	-0.27	**0.69**	0.56	1			**Y**	**Y**
Unit Stream Power (W m^{-2})	0.29	0.17	0.58	0.36	1		**N**	**N**
Mean Depth (cm)	0.17	-0.24	-0.1	-0.5	0.16	1	**Y**	**Y**
Mean Age (cal y BP)	0.66	**-0.73**	0.27	-0.41	0.11	0.27	1	
Sediment Flux (Mg 1000 yr^{-1} m^{-1})	-0.25	-0.5	-0.58	-0.66	-0.4	0.45	0.04	1

Pearson correlation coefficients (r) for potential predictors variables, mean radiocarbon ages, and estimated floodplain sediment mass flux per unit valley length of seven study reaches. Values in bold indicate correlations significant at the 90% confidence level ($p \leq 0.1$). Variables included (Y) are distinguished from variables excluded (N) in multiple regression for mean age and flux in the appropriate column.

Supplementary Table S4. Correlations for predictor variables of floodplain disturbance in the 2013 food.

	Elevation (m)	Cumulative downstream distance (m)	Drainage Area (km^2)	Channel Slope (m/m)	Valley Confinement (m/m)	Stream Power (W/m)	Unit Stream Power (W/m^2)	Change in Stream Power (W/m)	Change in Unit Stream Power (W/m^2)	Change in Valley Confinement (m/m)	Net Upstream Deposition (m^3)	Net Sediment Deposition (m^3)
Elevation (m)	1											
Cumulative downstream distance (m)	**-0.997**	1										
Drainage Area (km^2)	**-0.952**	0.962	1									
Channel Slope (m/m)	**0.263**	**-0.253**	**-0.321**	1								
Valley Confinement (m/m)	**0.233**	**-0.230**	-0.112	**-0.387**	1							
Stream Power (W/m)	**-0.206**	**0.217**	**0.168**	**0.849**	**-0.377**	1						
Unit Stream Power (W/m^2)	**-0.253**	**0.251**	**0.166**	**0.771**	**-0.561**	**0.906**	1					
Change in Stream Power (W/m)	-0.012	0.015	0.029	**0.315**	-0.044	**0.392**	**0.226**	1				
Change in Unit Stream Power (W/m^2)	0.005	0.001	0.012	**0.299**	**-0.181**	**0.346**	**0.379**	0.736	1			
Change in Valley Confinement (m/m)	0.012	-0.016	-0.015	-0.019	**0.462**	-0.006	-0.112	-0.072	**-0.357**	1		
Net Upstream Deposition (m^3)	**-0.201**	**0.220**	**0.300**	**-0.262**	**0.257**	-0.146	**-0.233**	-0.049	0.001	-0.144	1	
Net Sediment Deposition (m^3)	**-0.241**	**0.256**	**0.300**	**-0.252**	**0.261**	-0.137	**-0.230**	-0.020	-0.099	0.064	**0.693**	1

Pearson correlation coefficients (r) for potential predictor variables, net disturbed volume of sediment from the 2013 flood along 155 reaches on North Saint Vrain Creek. Values in bold indicate correlations significant at the 90% confidence level ($p \leq 0.01$). Source data are provided as a Source Data file. Source data are provided as a Source Data file.
